# Inflammation as a Risk Factor in Cardiotoxicity: An Important Consideration for Screening During Drug Development

**DOI:** 10.3389/fphar.2021.598549

**Published:** 2021-04-19

**Authors:** Chiara Campana, Rafael Dariolli, Mohamed Boutjdir, Eric A. Sobie

**Affiliations:** ^1^ Department of Pharmacological Sciences, Icahn School of Medicine at Mount Sinai, New York, NY, United States; ^2^ Cardiovascular Research Program, VA New York Harbor Healthcare System, Brooklyn, NY, United States; ^3^ Department of Medicine, Cell Biology and Pharmacology, State University of New York Downstate Medical Center, Brooklyn, NY, United States; ^4^ Department of Medicine, New York University School of Medicine, New York, NY, United States

**Keywords:** drug-induced cardiotoxicity, drug-induced arrhythmias, systemic inflammation, cardiac electrophysiology, quantitative systems pharmacology

## Abstract

Numerous commonly prescribed drugs, including antiarrhythmics, antihistamines, and antibiotics, carry a proarrhythmic risk and may induce dangerous arrhythmias, including the potentially fatal Torsades de Pointes. For this reason, cardiotoxicity testing has become essential in drug development and a required step in the approval of any medication for use in humans. Blockade of the hERG K^+^ channel and the consequent prolongation of the QT interval on the ECG have been considered the gold standard to predict the arrhythmogenic risk of drugs. In recent years, however, preclinical safety pharmacology has begun to adopt a more integrative approach that incorporates mathematical modeling and considers the effects of drugs on multiple ion channels. Despite these advances, early stage drug screening research only evaluates QT prolongation in experimental and computational models that represent healthy individuals. We suggest here that integrating disease modeling with cardiotoxicity testing can improve drug risk stratification by predicting how disease processes and additional comorbidities may influence the risks posed by specific drugs. In particular, chronic systemic inflammation, a condition associated with many diseases, affects heart function and can exacerbate medications’ cardiotoxic effects. We discuss emerging research implicating the role of inflammation in cardiac electrophysiology, and we offer a perspective on how *in silico* modeling of inflammation may lead to improved evaluation of the proarrhythmic risk of drugs at their early stage of development.

## Introduction

During drug development, the possibility of cardiotoxicity in the form of drug-induced Torsades de Pointes (TdP) must be evaluated for all new chemical entities. This is assessed by testing for potential block of the rapid delayed rectifier current, I_Kr_, and by measuring changes to the QT interval in healthy volunteers, under guidelines established by the International Conference on Harmonization and agreed to by regulatory bodies (Food and Drug Administration, HHS, 2005a; 2005b). More recently, initiatives such as CiPA, the Comprehensive *in vitro* Proarrhythmia Assay, aim to combine multiple preclinical measurements for a more complete picture of potential drug-induced arrhythmia (Colatsky et al., 2016; Vicente et al., 2018). Despite these requirements, many approved drugs are associated with isolated instances of TdP, and all drugs are monitored closely through databases such as the FDA Adverse Event Reporting System (Wysowski and Swartz, 2005; Poluzzi et al., 2012). In addition, preclinical efforts at cardiotoxicity testing focus primarily on how drugs influence the electrophysiology of healthy hearts, whereas TdP, when it occurs, is often associated with specific populations of patients, sometimes those with comorbidities (Roden, 2016; El-Sherif et al., 2018). In this perspective we discuss one such comorbidity, systemic inflammation, and argue that a careful consideration of inflammation’s effects on ventricular electrophysiology may enable a better understanding of when, and in what patient groups, particular drugs may increase TdP risk.

A PubMed search using the keywords *cardiotoxicity AND inflammation* reveals that 553 papers, of which 119 are reviews, have been published on this topic since 1976. In last 14 months alone, 134 articles were published, indicating increasing interest in the interactions between inflammation and drug-induced cardiac arrhythmias. The advent of Covid-19 provides perhaps the most recent evidence of how systemic inflammation is not only a determinant of disease severity, but also a factor that should be taken into account when choosing the preferred course of therapy (Lazzerini et al., 2020b). Drug repurposing, which has played a role in identifying treatment options for Covid-19 patients, has brought to public attention the intrinsic cardiac risk of some of the existing drugs on the market. Variability in a patient’s response to a disease, along with the innate complexity and differences in disease pathophysiology, can serve as obstacles in the establishment of the cardiotoxic risk of a given drug and render it difficult for clinicians to weigh the risk and benefit of therapeutics. In general, patients with underlying cardiac comorbidities, such as structural heart disease, are at higher arrhythmia risk, and factors such as age, electrolyte imbalance, and body weight also have an impact on drug-induced arrhythmia (Heist and Ruskin, 2010; Kannankeril et al., 2010, 2011). More recently, systemic inflammation, which is highly correlated with the occurrence of atrial fibrillation (Lazzerini et al., 2019a) and ventricular arrhythmia, has been implicated as a condition that can increase the risk of adverse cardiac events (Lazzerini et al., 2015b; Korantzopoulos et al., 2018).

An acute inflammatory response begins with activation of the immune system and the production of cytokines and other chemical mediators (Feghali-Bostwick and Wright, 1997; Zhang and An, 2007). Although the mediators that are produced depend on the nature of the inflammatory trigger, their general function is to stimulate target tissues to adjust to the new inflamed condition, including through the recruitment of cellular populations such as neutrophils and macrophages (Medzhitov, 2010; Remick, 2014). The release of cytokines such as interleukin-6 (IL-6), tumor necrosis factor-α (TNF-α) and interleukin-1β (IL-1β) during the inflammatory process can also induce systemic changes that include the production of C-reactive protein (CRP) by the liver and the production of proinflammatory prostaglandins in the brain (Medzhitov, 2010). Because CRP levels correlate with the levels of cytokines, they are utilized as a surrogate marker for monitoring the latter and have been adopted by clinicians to assess disease severity and prognosis (Osman et al., 2006). Inflammation is often triggered by viral and bacterial infections and resolves quickly once the infectious agents have been controlled. However, in the face of a persistent infection or when the inflammatory source is further enhanced through a positive feedback loop, chronic inflammation can arise (Medzhitov, 2010). This can also occur in conditions such as obesity and neurodegenerative disease (Medzhitov, 2010; Nathan and Ding, 2010).

Cytokines that are released during acute and chronic inflammation may influence the function and expression of cardiac ion channels, thereby potentially explaining the increased risk of arrhythmia and sudden cardiac death in the presence of inflammation (Lazzerini et al., 2015b; 2020a). Investigators have begun to uncover the pathophysiology behind this arrhythmogenicity by using patients with chronic inflammatory disorders to better understand the effects of inflammation on specific cardiac ion channels (Alí et al., 2018). In light of this developing research, the term inflammatory cardiac channelopathies has been proposed (Lazzerini et al., 2018; 2019b). For instance, Aromolaran et al. (2018) have recently shown that elevated serum inflammatory markers will inhibit the human ether-à-go-go-related gene (hERG) channel, reducing the rapid delayed rectifier current (I_Kr_) and leading to action potential (AP) prolongation. This and similar recent results have prompted a consideration of how endogenous inflammation and potential proinflammatory effects of drugs may affect preclinical cardiotoxicity studies.

We propose that evaluating the effects of inflammation on cardiac electrical function is especially important when testing the cardiotoxicity of: 1) drugs that induce inflammation as a side effect, and 2) drugs that are used to treat diseases that are associated with chronic inflammation.

## Inflammation and Cardiotoxicity Testing

### Inflammation and the Heart

Acute inflammation is determined by defense mechanisms that activate the immune system and transiently respond to a pathogen, or other triggers, in ways that generally benefit the organism. However, systemic chronic inflammation has a deleterious effect on the body (Medzhitov, 2010). It can be caused by obesity, viral or microbial infection, autoimmune disease, or cancer (Berg and Scherer, 2005; Koene et al., 2016). Heart failure with preserved ejection fraction (HFpEF) is responsible for roughly half of heart failure cases and has a wide variety of underlying causes (Shah et al., 2016; Packer and Kitzman, 2018). Obesity, diabetes and hypertension are common comorbidities in patients with HFpEF, and research in the past decade has recognized their increasingly important role in the pathophysiology of HFpEF, such that an obesity-associated HFpEF phenotype has been defined (Paulus and Tschöpe, 2013; Shah et al., 2016; Packer and Kitzman, 2018). In the obese HFpEF phenotype, obesity-driven systemic inflammation leads to cardiomyocyte stiffness and interstitial fibrosis that contribute to heart failure development (Paulus and Tschöpe, 2013; Shah et al., 2016; Oh et al., 2019).

With respect to electrophysiology, QT interval is generally prolonged in individuals with systemic inflammation and can be correlated in some instances with CRP levels and the incidence of sudden death (Kazumi et al., 2003; Hussein et al., 2013). For example, patients suffering from rheumatoid arthritis, an autoimmune disease, are at higher risk of experiencing cardiovascular diseases and sudden death (Lazzerini et al., 2015a). In this study, the release of proinflammatory cytokines was one factor that accounted for QT interval prolongation and the risk of sudden death, and the immunosuppressive drug tocilizumab returned the QT interval to a normal value within three months of treatment. Tocilizumab, which binds to the IL-6 receptor, is thought to work by inhibiting both IL-6 signaling and the release of additional cytokines such as TNF-α. The inflammatory pathway common to rheumatoid arthritis and several other conditions leads to structural remodeling of cardiac tissue, inducing fibrosis and coronary atherosclerosis, as well as electrical remodeling that affects the function and the expression of cardiac ion channels. Importantly, the study by Lazzerini et al. (2015a) emphasizes the role of inflammation-induced QT prolongation in precipitating cardiovascular complications often assumed to be secondary to the altered cardiovascular structure.

In isolated cardiomyocytes, the AP is a surrogate for the ECG such that a prolonged cellular AP duration (APD) corresponds to longer QT interval on the ECG, and a higher propensity for early after depolarizations, which can trigger ventricular arrhythmias (Wit, 2018). Because the AP is determined by the balance between inward depolarizing currents and outward hyperpolarizing currents, drugs or genetic mutations that, reduce the magnitude of K^+^ currents or increase the magnitude of Ca^2+^ currents can cause an increase in the APD. Although the connection between inflammation and QT prolongation is not completely understood, proinflammatory cytokines TNF-α, IL-6 and IL-1β have been shown to alter both the function of ion channels and their levels of expression, in addition to the cardiovascular structural remodeling caused by inflammation (Lazzerini et al., 2017). We provide a summary of the relevant literature with a focus on the different time scales on which the remodeling occurs.

In terms of direct effects on cardiac ion channels, both IL-6 and TNF-α have been shown to inhibit hERG currents in HEK293 cells (Wang et al., 2004; Aromolaran et al., 2018), and IL-6 both inhibits hERG and prolongs APs in guinea pig hearts (Aromolaran et al., 2018). Acute exposure to IL-1β contributes to increased Ca^2+^ current density and longer APD in guinea pig ventricular cells (Li and Rozanski, 1993). Similarly, Hagiwara et al. (2007) showed that IL-6 rapidly increases Ca^2+^ current density and intracellular Ca^2+^ transients in mouse ventricular cells.

The longer-term effects of these cytokines have also been investigated. Wang et al. (2004) confirmed the proarrhythmic impact of TNF-α in isolated canine cardiomyocytes and showed time-dependent inhibitory effects on I_Kr_ such that APD values after 10 h of exposure were longer than those after 10 min of exposure. IL-6 also reduced hERG expression in HEK293 cells and adult guinea pig ventricular myocytes (Aromolaran et al., 2018). Consistent with these results, Monnerat et al. (2016) showed that rodent and human cells incubated for 24 h with IL-1β had reduced repolarizing transient outward K^+^ current (I_to_), increased Ca^2+^ spark frequency, and increased Ca^2+^/calmodulin-dependent protein kinase II (CaMKII) oxidation, which in turn intensifies Ca^2+^ leakage from the sarcoplasmic reticulum into the cytosol (Szekely and Arbel, 2018).

Over an even longer period of time, the three cytokines have been determined to alter myocardial contractility, which is consistent with the clinical presentation of chronic inflammation. When administered chronically, IL-6 has been shown to decrease cardiac contractility and induce cardiac hypertrophy in both mouse and human studies (Fontes et al., 2015). Combes et al. (2002) found that neonatal rat cardiac myocytes exposed to IL-1β for three days had reduced amplitude and maximum speed of contraction. Overexpression of TNF-α in mice induced decreased ejection fraction, atrial and ventricular arrhythmias, and heart failure (Feldman et al., 2000; Petkova-Kirova et al., 2006). Underlying these events is a reduction in I_to_ and delayed rectifier currents, accompanied by diminished Kv4.2, Kv4.3 and Kv1.5 protein expression (Petkova-Kirova et al., 2006).

### *In silico* Evaluation of Drug Safety

Decades after the first evidence of drug-induced TdP was discovered during treatment of atrial fibrillation with quinidine (Arthur and Wray, 1964), it is now well established that a wide range of prescription drugs carry a cardiotoxic risk. During drug development in the pharmaceutical industry, a new compound’s proarrhythmic potential is investigated early, to attempt to prevent drugs with high cardiotoxicity risk from ultimately entering the market. The most severe forms of acquired arrhythmias are often precipitated by drugs whose mechanism of action involves blockade of I_Kr_. The proarrhythmic risk associated with these drugs varies between individuals and depends on factors such as age, genetic background, and the presence of comorbidities or concomitant drug treatments (Polak et al., 2015). Due to the extensive usage of some of these medications individually or in combination, even a small proarrhythmic risk can be of concern. Moreover, due to the phenotypic differences among the individuals using these medications, it is difficult but imperative to identify who is at higher risk of developing life-threatening drug-induced arrhythmias.

Although block of I_Kr_, the so-called “hERG current”, has long been considered the simplest way of assessing a compound’s cardiotoxic risk, the limitations of this approach have led to newer initiatives such as CiPA (Sager et al., 2014). CiPA proposes to identify reliable proarrhythmia metrics by analyzing how drugs interact with a wide range of ionic channels, and by combining *in vitro* with *in silico* studies (Sager et al., 2014; Colatsky et al., 2016; Li et al., 2020). A multiscale *in silico* approach is adopted to evaluate the effect of channel blockade on the ventricular cardiomyocyte AP (Li et al., 2019). The *in silico* predictions are then validated in hiPSC-CMs. There has been tremendous progress in expanding the proarrhythmic risk evaluation to different spatial scales and correlating the events happening at the molecular level to the phenomena occurring at the organ level (Yang et al., 2020). The introduction of machine learning approaches and the definition of new metrics other than hERG blockade have helped accomplish a better stratification of drug-induced cardiotoxicity (Lancaster and Sobie, 2016).

While these efforts have certainly improved drug risk prediction, the majority of *in silico* cardiotoxicity studies, which have used models of healthy cardiomyocytes, have largely left unexplored how disease conditions may affect cardiotoxicity predictions (Chi, 2013; Li et al., 2017; Passini et al., 2017; Margara et al., 2020; Yang et al., 2020). Although it is important to study and understand these mechanisms, most drugs are used in individuals whose cardiac electrophysiology differs from that seen in healthy subjects. Models of pathological cardiac electrophysiology have been developed over the years to describe conditions such as heart failure or congenital heart diseases, such as long QT syndromes, caused by cardiac ion channel gene mutations (Niederer et al., 2019). Some cardiotoxicity studies have also explored the effect of these diseases on *in silico* predictions, recognizing that modeling pre-existing cardiovascular disorders may improve risk stratification (Christophe and Crumb, 2019; Llopis et al., 2019). However, only a limited number of diseases have been modeled, and the field is lacking a comprehensive study evaluating how the response to drugs differs between healthy and diseased human ventricular cardiomyocytes. Since inflammation: 1) underlies or occurs secondarily to many pathologies, and 2) has both direct and indirect effects on multiple cardiac electrophysiology mechanisms, it becomes imperative to explore this emerging role of inflammation in greater detail. We suggest that integrating inflammation in multiscale cardiac electrophysiology modeling would represent a first important step toward disease-specific cardiotoxicity studies.

### New Directions in Cardiotoxicity Testing

**FIGURE 1:**
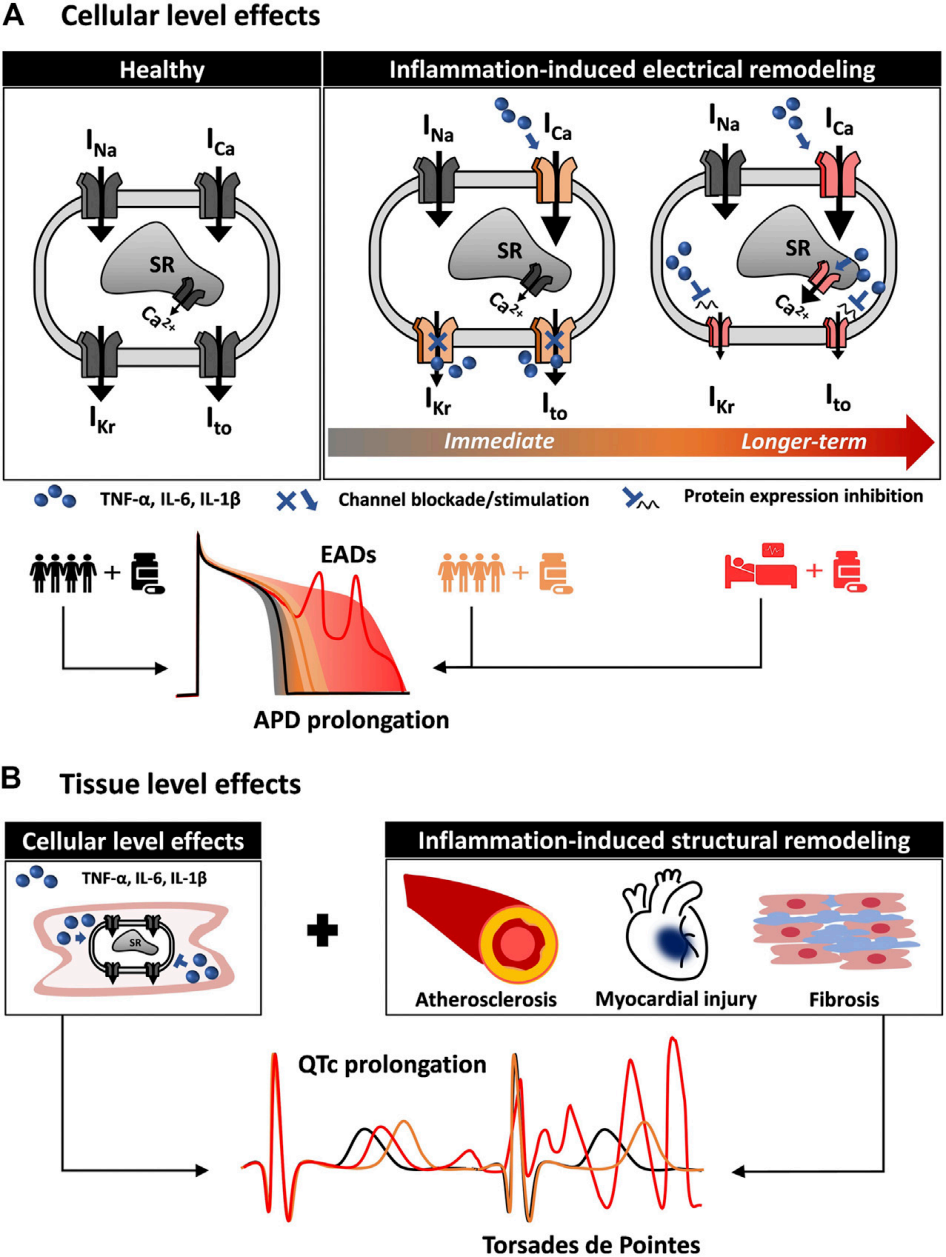
Multiscale quantitative systems pharmacology to integrate inflammation modeling into *in silico* drug safety evaluation. **(A)** Inflammation induces cardiac electrical remodeling. Proinflammatory cytokines can alter cardiac electrophysiology by interacting directly with the cardiac ion channels. By altering the normal function (immediate effect) and expression of cardiac ion channels (longer-term effect), inflammation is a source of variability in the cardiomyocyte response to the administration of drugs. In addition to variability among healthy individuals (black line and gray shaded area), the inflammatory state (orange and red) can profoundly alter the cellular pro-arrhythmia risk metrics normally evaluated in *in silico* drug safety. **(B)** Inflammation affects cardiac tissue pro-arrhythmia risk metrics. Inflammation also contributes to cardiac structural remodeling, affecting cardiac propagation and creating a proarrhythmic substrate. Together with the effects on the single cardiomyocyte, this can result in altered (orange and red) QT interval on the ECG.

Multiscale cardiac electrophysiology modeling allows for the study of drug interaction with cardiac ion channels. Several approaches have been previously used to achieve this, including simple pore block models and more complicated Markov chain models (Wiśniowska et al., 2014; Gando et al., 2020). The effects of proinflammatory cytokines on cardiac ion channels, such as the blockade of hERG by IL-6 described by Aromolaran et al. (2018), can be readily incorporated in cardiac electrophysiology modeling to more accurately stratify arrhythmic risk in patients based on their inflammatory state. Figure 1 shows a schematic of how integrating the effect of comorbidities, in particular inflammation, can alter cellular proarrhythmic markers. The first aspects to be included in *in silico* cardiotoxicity studies would be the direct actions of cytokines on cardiac ion channels combined with the indirect effects caused in myocytes by prolonged cytokine exposure (Figure 1A). Although effects of cytokines on certain ion channels have been demonstrated, as described above, it would be important to complement these with measurements of how the cytokines influence action potentials and intracellular [Ca^2+^] to infer whether additional effects might also contribute. Furthermore as more data become available, effects of cytokines can be incorporated into integrative models that combine electrophysiology and Ca^2+^ signaling with, for instance, cellular energetics (Gauthier et al., 2012) or intracellular signaling (Heijman et al., 2011; Gong et al., 2020). Inclusion of these features would allow for evaluation of how inflammation directly modifies the single cardiomyocyte behavior.

In a second step, structural remodeling can also be integrated into computational analyses if a cardiac tissue is modeled (Figure 1B). For instance, inflammation-induced fibrosis affects cardiac propagation, and models of fibroblasts interconnected with cardiac myocytes have been previously implemented (Zeigler et al., 2016). Importantly, hiPSC-CMs can serve as a valuable platform to support the development of accurate models of inflammation-induced electrical remodeling. The effect of many proinflammatory cytokines, alone or in combination, can be evaluated in such systems to inform parameters (such as channel conductance) of *in silico* human models (Yücel et al., 2017). Computational approaches (Gong and Sobie, 2018) have been recently proposed to address the limitations stemming from the immature phenotype of hiPSC-CMs. This can facilitate the translation of recent findings (Yücel et al., 2017) and future discoveries from hiPSC-CMs to human adult cardiomyocytes.

Variability in drug response among healthy individuals is important in drug development and drug cardiotoxicity studies. Cardiac electrophysiological heterogeneity plays an important role in current *in silico* cardiotoxicity studies, and population-based modeling has become an integral aspect of computational cardiac electrophysiology (Sarkar et al., 2012; Britton et al., 2013; Muszkiewicz et al., 2016; Ni et al., 2018). Variability in a healthy population of individuals is typically included in cardiomyocyte models by randomly varying, within a range of physiological values, the ion channels’ conductance and gating variables (Sarkar et al., 2012). Age, cardiac disease, and biological sex are important factors that influence electrophysiology (Bednar et al., 2002; Yang et al., 2017), and these can be incorporated into simulations of cardiotoxicity risk (Varshneya et al., 2021). In a computational framework that accounted for inflammation, it would be important to incorporate variability in both the healthy and chronic inflammation populations, since inflammation itself may influence the factors that contribute to arrhythmia risk.

We postulate that in addition to considering variability across populations of healthy individuals, *in silico* cardiotoxicity studies should begin to integrate more detailed modeling of disease pathways. Medications labeled as safe might compromise the cardiac function in patients already at higher risk of cardiac complications because of inherent comorbidities. Although certain disease processes may have little influence, chronic diseases, viral and bacterial infections, and cancer are likely to disturb both normal cardiac function and how the heart interacts with medications. Potentially proarrhythmic drugs, such as the widely prescribed antibiotic clarithromycin, the cancer medication tamoxifen and the tyrosine kinase inhibitor vandetanib, used for treatment of thyroid cancer, are all used in patients with altered electrophysiology. The pathological profile of conditions such as cancer and microbial infections includes increased proinflammatory cytokine levels that, as described in the previous sections, are known to significantly alter many physiological cardiac mechanisms. Considering the impact of inflammation on cardiac ion channel remodeling would be particularly important in these cases and would signify an important step towards incorporating disease modeling into *in silico* drug safety testing.

Despite the complexity of the mechanisms underlying systemic inflammation, it is a phenomenon that should be explored more in depth in the field of computational cardiac electrophysiology. An increasing amount of research has characterized the interplay among systemic inflammation and long QT syndrome and arrhythmogenicity. The multitude of diseases where inflammation plays a role makes it an important process to evaluate in studies of drug response and cardiotoxicity.

## Data Availability

The original contributions presented in the study are included in the article/Supplementary Material, further inquiries can be directed to the corresponding author.
